# Genomic prediction based on data from three layer lines using non-linear regression models

**DOI:** 10.1186/s12711-014-0075-3

**Published:** 2014-11-06

**Authors:** Heyun Huang, Jack J Windig, Addie Vereijken, Mario PL Calus

**Affiliations:** Animal Breeding and Genomics Centre, Wageningen UR Livestock Research, PO Box 338, 6700 AH Wageningen, The Netherlands; Hendrix Genetics, Research, Technology & Services BV, PO Box 114, 5830 AC Boxmeer, The Netherlands

## Abstract

**Background:**

Most studies on genomic prediction with reference populations that include multiple lines or breeds have used linear models. Data heterogeneity due to using multiple populations may conflict with model assumptions used in linear regression methods.

**Methods:**

In an attempt to alleviate potential discrepancies between assumptions of linear models and multi-population data, two types of alternative models were used: (1) a multi-trait genomic best linear unbiased prediction (GBLUP) model that modelled trait by line combinations as separate but correlated traits and (2) non-linear models based on kernel learning. These models were compared to conventional linear models for genomic prediction for two lines of brown layer hens (B1 and B2) and one line of white hens (W1). The three lines each had 1004 to 1023 training and 238 to 240 validation animals. Prediction accuracy was evaluated by estimating the correlation between observed phenotypes and predicted breeding values.

**Results:**

When the training dataset included only data from the evaluated line, non-linear models yielded at best a similar accuracy as linear models. In some cases, when adding a distantly related line, the linear models showed a slight decrease in performance, while non-linear models generally showed no change in accuracy. When only information from a closely related line was used for training, linear models and non-linear radial basis function (RBF) kernel models performed similarly. The multi-trait GBLUP model took advantage of the estimated genetic correlations between the lines. Combining linear and non-linear models improved the accuracy of multi-line genomic prediction.

**Conclusions:**

Linear models and non-linear RBF models performed very similarly for genomic prediction, despite the expectation that non-linear models could deal better with the heterogeneous multi-population data. This heterogeneity of the data can be overcome by modelling trait by line combinations as separate but correlated traits, which avoids the occasional occurrence of large negative accuracies when the evaluated line was not included in the training dataset. Furthermore, when using a multi-line training dataset, non-linear models provided information on the genotype data that was complementary to the linear models, which indicates that the underlying data distributions of the three studied lines were indeed heterogeneous.

**Electronic supplementary material:**

The online version of this article (doi:10.1186/s12711-014-0075-3) contains supplementary material, which is available to authorized users.

## Background

Genomic estimated breeding values (GEBV) are generally predicted by a regression model [1] trained by a set of animals with known phenotypes and genotypes for a dense marker panel that covers the genome [2]. Prediction accuracy of such models depends on several factors, among which size of the set of training animals is most important, which has been addressed in several studies [2,3] that consistently claim that the biggest limitation for the accuracy of genomic prediction of livestock is the number of animals with both genotype and phenotype data. In most cases, the number of markers is however substantially larger than the number of training samples. This means that genomic prediction typically has a small sample-to-size ratio, which is also known as a *n* << *p* problem [1]. One of the major disadvantages is that *n* << *p* may lead to a severe over-fitting problem, which may affect the accuracy of the predictions in a validation dataset. Dimension reduction [4,5] could be an alternative approach to retain the most relevant information of the genotype data [6,7] in a low-dimensional vector space.

Our study aimed at investigating a more straightforward and feasible approach to alleviate the *n* < < *p* problem, which consists of enlarging the training set by using data from multiple populations. However, studies on across-breed genomic prediction using 50 k genotypes have shown that the use of a multi-breed training dataset typically results in a limited or no increase in accuracy compared to using training data from a single breed [8-11]. Previous studies have hypothesized that in order to successfully combine training datasets of Holstein-Friesian and Jersey dairy cattle breeds, genotypes on at least 300 000 SNPs (single nucleotide polymorphisms) should be used [12].

Besides insufficient SNP density, another reason that may explain the limited increase in prediction accuracy observed when using multi-population compared to single-population training data could be that the commonly used models cannot deal appropriately with heterogeneous multi-population data. To date, all across-population genomic prediction studies have used linear models. These linear models generally assume that the effect of a SNP in one population is the same in another population. This assumption can be violated due to several reasons. First, the linkage disequilibrium (LD) may differ between populations. Second, it is quite likely that at least some of the segregating QTL (quantitative trait loci) are population-specific. Third, the absolute effect of a QTL may differ between populations because of differences in genetic background. The assumption of linearity may be too rigorous for any of these situations, especially when using the common 50 k SNP chip. In fact, if differences between populations or lines are too large, predictive ability of across-breed genomic prediction with linear models may be lower than that of within-breed genomic prediction [13]. A few studies have proposed to use multi-trait linear models [14-16], where trait by line combinations are modelled as separate but correlated traits, to try to accommodate these issues.

As an alternative solution, we propose to use non-linear models by kernel learning [13,17,18]. The basic idea is to predict the breeding value of a test animal using a limited number of training animals with similar genotypes that do not necessarily come from a single population. By doing so, the entire heterogeneous data space spanned by genotypes is decomposed into a large number of locally homogeneous sub-areas [19-21], regardless of their population of origin. Such a model might be able to extract the useful information across populations. At the very least, the non-linear models by kernel learning are expected to better capture the heterogeneous nature of the data compared to linear models.

The objective of this study was to investigate the accuracy of multi-line genomic prediction using non-linear models by kernel learning and a linear model that modelled trait by line combinations as separate but correlated traits, and to compare the prediction accuracy of these models to that of commonly used linear genomic prediction models presented by Calus et al. [22]. This comparison was performed with a dataset that included three lines of layer hens.

## Methods

### Linear regression

Linear regression models [23] have been widely used to implement genomic prediction [24]. In concrete terms, the ultimate goal of a regression task is to predict an unseen value *y* from a vector of observations/features **x**. In the scenario of genomic prediction, (**x**,*y*) corresponds to genotypes (**x**) and phenotypes (*y*) of *n* training individuals. Linear regression uses a linear function to map the observations **x** to the responsible value *y* by a vector **w** as the linear weights on **x**:

1 $$ y={\mathbf{w}}^t\mathbf{x}, $$

where the weight vector **w** can be estimated using the training data. To best approximate the underlying functional relationship between **x** and *y* by Equation (1), ridge regression aims at minimizing the average quadratic loss (*L*) between the true response value *y*_*i*_ and **w**^*t*^**x**_*i*_:

2 $$ L\left(\mathbf{w}\right)={\displaystyle {\sum}_{i=1}^n{\left({y}_i-{\mathbf{w}}^t{\mathbf{x}}_i\right)}^2}+\gamma {\left|\left|\mathbf{w}\right|\right|}^2\equiv {\left|\left|\mathbf{y}-\mathbf{X}\mathbf{w}\right|\right|}^2+\gamma {\left|\left|\mathbf{w}\right|\right|}^2\ . $$

The vector **y** refers to a column vector [*y*_1_, *y*_2_ , …, *y*_*n*_ ]^*t*^ that contains the phenotypes of all training animals, while the matrix **X** contains the genotypes of all training animals. The norm of **w** is the regularization term. Adding it into the objective function alleviates the over-fitting problem, which might be detrimental to prediction performance since the number of genotypes is generally much larger than the number of training samples. Parameter *γ* refers to the weight given to the regularization term.

Minimization of the loss function *L* by Equation (2) with regard to **w** results in the following estimate:

3 $$ {\mathbf{w}}^{*}={\left({\mathbf{X}}^t\mathbf{X}+\gamma \mathbf{I}\right)}^{-1}{\mathbf{X}}^t\mathbf{y}. $$

If the following matrix lemma [25] is applied:

$$ {\left({\mathbf{A}}^{-1}+{\mathbf{B}}^t{\mathbf{D}}^{-1}\mathbf{B}\right)}^{-1}{\mathbf{B}}^t{\mathbf{D}}^{-1}=\mathbf{A}{\mathbf{B}}^t{\left(\mathbf{BA}{\mathbf{B}}^t+\mathbf{D}\right)}^{-1}, $$

the solution to w* can be reformulated to:

4 $$ {\mathbf{w}}^{*}={\mathbf{X}}^t{\left(\mathbf{X}{\mathbf{X}}^t+\gamma \mathbf{I}\right)}^{-1}\mathbf{y}\ . $$

With this estimate, the prediction y* based on the test vector **x**_*t*_ becomes:

5 $$ {y}^{*}={\mathbf{w}}^{*t}{\mathbf{x}}_t={\mathbf{y}}^t{\left(\mathbf{X}{\mathbf{X}}^t+\gamma \mathbf{I}\right)}^{-1}\mathbf{X}{\mathbf{x}}_t. $$

These descriptions provide the basis for the development of the non-linear models presented below. For comparison, we included two linear models, i.e. ridge-regression based on principal component analysis (RRPCA) and genome-enabled best linear unbiased prediction (GBLUP) [26]. More detailed descriptions of these models, and the results obtained with these models on this data, are in [22].

### Multi-trait genome-enabled best linear unbiased prediction (MTGBLUP)

One of the disadvantages of linear regression is that the underlying data structures might not be well characterized by the linear weights. In genomic prediction, this implies that the estimated effects are not necessarily strictly additive genetic effects [17], and in the context of multi-breed genomic prediction, this may be further interpreted as the true SNP effects not being the same in different breeds or lines. One straightforward approach to allow estimated SNP effects to differ between lines, is to use a multi-trait GBLUP (MTGBLUP) model that allows genetic correlations between the lines to differ from 1 [14]. The data available was not large enough to estimate these correlations; however, additional data was available on non-genotyped animals for each line. Therefore, pairwise genetic correlations between lines were estimated by applying REML (restricted maximum likelihood) [27] with a model that used the inverse of a combined pedigree and genomic relationship matrix [28] that included all three lines. Using this combined relationship matrix, the number of training records ranged from 24 906 to 27 896 across the three lines, while when only genotyped animals were considered, it ranged from 1004 to 1023. Using the estimated variance components, the MTGBLUP model was run using a G-matrix as described in [26], such that only genotyped animals were included in the reference population.

### Non-linear kernel regression

Another interpretation of the expectation that the underlying data structures across breeds or lines might not be well characterized by the linear weights is that the inherent mapping function might not be linear. To capture such data features, the common tandem is to adopt a non-linear function (·) {**x** → *φ*(**x**)}. The non-linear function results in new representations of genotypes that may be associated with both additive and non-additive effects [17,29]. Accordingly, Equation (5) can be modified by replacing **x** by *φ*(**x**):

6 $$ {y}^{*}={\mathbf{y}}^t{\left(\boldsymbol{\varPhi} \left(\mathbf{X}\right)\boldsymbol{\varPhi} {\left(\mathbf{X}\right)}^t+\gamma \mathbf{I}\right)}^{-1}\boldsymbol{\varPhi} \left(\mathbf{X}\right)\varphi \left({\mathbf{x}}_t\right), $$

where ***Ф***(**X**) contains the transformed genotypes using *φ*(**x**). Interestingly, the predictor does not necessarily depend on the mapping function *φ*(**x**) but on the inner products between the vectors *φ*(**x**) and *φ*(**y**), namely *φ*(**x**)*φ*(**y**)^*t*^, as a result of the following terms in (6):

- **Ф**(**X**)**Ф**(**X**)^*t*^: the element of the resultant matrix on the *i*th column and *j*th row is *φ*(**x**_*i*_)*φ*(**x**_***j***_)^*t*^,
- **Ф**(**X**)*φ*(**x**_*t*_): the *i*th element of the resultant vector is *φ*(**x**_*i*_)*φ*(**x**_*t*_)^*t*^.

This property implies that the design of the kernel function *K*(**x**, **t**) = *φ*(**x**)*φ*(**t**)^*t*^ is sufficient to give rise to the predictor without any knowledge on the mapping function *φ*(**x**):

7 $$ {y}^{*}={\mathbf{y}}^t{\left(\mathbf{K}+\gamma \mathbf{I}\right)}^{-1}\mathbf{k}, $$

where **K** is a matrix with elements *K*(**x**_*i*_, **x**_*j*_), *i*, *j* = 1, 2, …, *n* and **k** is a vector with elements *K*(**x**_*i*_, **x**_*t*_), *i* = 1, 2, …, *n*.

#### Construction of kernels

One possible interpretation of kernel learning is that the kernel function of two vectors **x** and **t**, *K*(**x**, **t**), to some extent describes the similarity between **x** and **t** by tending to yield a relatively large value when **x** is similar to **t**. There are two typical approaches to evaluate the similarity of two vectors: cross-correlation **x**^*t*^**t** and distance *d*(**x**, **t**). Both of these are intrinsically related: **x**^*t*^**t** is inversely proportional to *d*(**x**, **t**) if the measure *d* is Euclidean distance: *d*(**x**, **t**) = **||x** − **t||**^2^ = **||x||**^2^ + **||t||**^2^ − 2**x**^*t*^**t**. Therefore, in this study both cross-correlation-based kernels [13,30] and distance-based kernels [30-33] that use those two similarity measures were used.

##### Cross-correlation based kernels

The polynomial kernel is the most classical cross-correlation-based kernel [28,34] that depends on the inner product of two vectors:

8 $$ K\left(\mathbf{x},\mathbf{t}\right)={\left({\mathbf{x}}^t\mathbf{t}\right)}^l. $$

This kernel maps the original feature space into one that is spanned by monomials of degree *l*. A more general definition of the polynomial kernel is:

9 $$ K\left(\mathbf{x},\mathbf{t}\right)={\left({\mathbf{x}}^t\mathbf{t}+\mathbf{c}\right)}^l, $$

which is called an inhomogeneous polynomial kernel since a unit shift is added onto the inner product of two vectors. Compared with the homogeneous kernel given by Equation (8), the explicit mapping function of this kernel contains all monomials whose degrees are equivalent to or smaller than *l*.

##### Distance-based kernels

Similarity can also be measured by the distance *d*: if **x** and **t** are similar, the function value of *d*(**x**, **t**) should be small. Mathematically speaking, the distance function should satisfy the following three properties:

1. *d*(**x**, **x**) ≥ **0**,
2. *d*(**x**, **t**) = *d*(**t**, **x**),
3. *d*(**x**, **t**) < *d*(**x**, **z**) + *d*(**z**, **t**).

Then, a valid kernel can be constructed by the following equation:

10 $$ K\left(\mathbf{x},\mathbf{t}\right)={e}^{-d\left(\mathbf{x},\mathbf{t}\right)}. $$

Distance-based kernels are derived from *L*_*p*_-norm distance, which has been proven to satisfy the aforementioned requirements [34]:

$$ ||\mathbf{x}{||}_p={\left({x}_1^p+{x}_2^p+\dots +{x}_m^p\right)}^{\frac{1}{p}}. $$

Two well-known distance kernels are special cases of this general equation: the radial basis function (RBF) kernel (*p* = 2, also known as Gaussian kernel) [31] and the Laplacian Kernel (*p* = 1) [33]:

$$ {K}_G\left(\mathbf{x},\mathbf{t}\right)={\boldsymbol{e}}^{-||\mathbf{x}-\mathbf{t}{||}_2}, $$

$$ {K}_L\left(\mathbf{x},\mathbf{t}\right)={\boldsymbol{e}}^{-||\mathbf{x}-\mathbf{t}{||}_1}. $$

### Comparison of methods

In our study, accuracy of genomic prediction based on multi-line training was evaluated for two non-linear models that were based on two different kernels that are the most representative of the two categories of kernels described in the previous section [35]. The first uses the RBF kernel and is termed “RBF” hereafter, and the second uses the polynomial kernel and is termed “Poly” hereafter. Linear regression, also known as ridge regression (RR), is a special case of kernel linear regression that adopts the linear kernel [13]. A method equivalent to RR, i.e. GBLUP that uses a genomic relationship matrix [26], is applied here for comparison.

Considering that the number of SNPs is relatively large compared to the number of animals with phenotypes, all models were also implemented after performing principal component analysis (PCA) to reduce the data dimensions while still explaining 97% of the variance of the SNP genotypes in the data. These three models are termed RRPCA for RR, RBFPCA for RBF kernel based linear regression and PolyPCA for polynomial kernel based linear regression.

### Data, pre-analysis, and experimental configurations

To compare the models, data of two brown and one white lines of layer chickens were analysed. The brown layer lines B1 and B2 were closely related to each other, while the white line (W1) was only distantly related to the brown lines. The phenotype data used was the number of eggs in the first production period until the hens reach the age of 24 weeks. Across the three lines, 3753 female birds had both phenotypes and genotypes for 45 974 SNPs from the chicken 60 k Illumina Infinium iSelect Beadchip [36] after editing. More details on the dataset and on the editing of the SNP data are described in Calus et al. [22].

Seven different training sets and one validation set per line were defined to evaluate the accuracy of genomic prediction with single- and multi-line training datasets. For each line, the youngest generation, containing 238 to 240 birds, was used as a validation set. Breeding values for the validation animals were predicted using phenotypes of the training set, which were pre-corrected for hatch week. For the validation animals, the correlation coefficient between the GEBV and their observed phenotypes were computed to evaluate the accuracy of genomic prediction with various training datasets. These correlations are hereafter referred to as ‘predictive correlations’. Commonly, such correlations are divided by the square root of the heritability of the trait to reflect the accuracies of predictions of true breeding values. In this case, we did not do that, because such an adjustment assumes that all the captured genetic variance is additive, while the kernel functions may capture some non-additive effects. Approximate standard errors of the predictive correlations were computed using the expected sampling variance of an estimated correlation ($\widehat{\rho}$), as $\frac{1-{\widehat{\rho}}^2}{\sqrt{N-2}}$ where *N* is the number of training animals [24]. The coefficient of the regression of phenotypes on GEBV (*b*_1_) was computed to evaluate bias of the predictions. Standard errors of the regression coefficients, denoted as $S{E}_{b_1}$, were derived with bootstrapping, which involved computing regression coefficients for 10 000 bootstrapping samples of the 238 to 240 validation animals, using the R-package “boot” [37]. The regression coefficients were considered as not significantly different from 1 when $\left|{b}_1-1\right|<2\times S{E}_{b_1}$ [38].

The first three training sets consisted of one of the three lines. The next three training sets included each of the three pairwise combinations of the three lines. The last training set included layers from all three lines. The resulting training sets included ~1000 to 3000 animals, and the number of segregating SNPs ranged from 30 508 to 45 974 [22].

## Results

### Genetic correlations between lines

The estimated genetic correlations between the three lines are in Table 1. The genetic correlation between lines B1 and B2 was equal to 0.63, thus significantly larger than 0, which confirms that B1 and B2 are closely related lines. Genetic correlations between lines B1 and W1 and between lines B2 and W1 were equal to −0.26 and −0.55, respectively. The large standard errors of these estimates show that the estimated genetic correlation between line B1 and W1 is not significantly different from 0, while the correlation between B2 and W1 is significantly lower than 0.

**Table 1:** **Estimated genetic correlations between egg production in the three layer lines (standard errors in brackets)**

**Line**	**B2**	**W1**
B1	0.63 (0.14)	−0.26 (0.37)
B2		−0.55 (0.37)

### Accuracy of genomic predictions

Tables 2, 3 and 4 show the predictive correlations for each line of six methods using seven training datasets. In the following, we first describe results across the training datasets and then differences between the methods.

**Table 2:** **Performance comparison of seven prediction methods in seven training scenarios for line B1**

	**Training data**						
**Model**	**B1**	**B2**	**W1**	**B1 + B2**	**B1 + W1**	**B2 + W1**	**B1 + B2 + W1**
GBLUP^1^	0.322	0.182	−0.033	0.316	0.306	0.149	0.304
RRPCA^1^	0.286	0.147	0.064	0.280	0.279	0.156	0.276
MTGBLUP	0.282	0.194	−0.037	0.293	0.274	0.190	0.292
Poly	0.281	−0.026	0.013	0.281	0.283	0.008	0.283
PolyPCA	0.280	−0.046	0.013	0.280	0.282	0.007	0.282
RBF	0.315	0.206	0.006	0.321	0.315	0.204	0.321
RBFPCA	0.281	0.128	0.029	0.285	0.281	0.129	0.285

The predictive correlation is computed as the correlation coefficient of the predicted value and phenotype of line B1; GBLUP: genome-enabled best linear Unbiased Prediction; RRPCA: ridge regression principal component analysis; MTGBLUP: multi-trait GBLUP; Poly: polynomial kernel based linear models; RBF: radial basis function kernel based linear models; RR/Poly/RBF-PCA: the model with the features reduced by PCA.

Approximated SE across the genomic prediction models and training data sets ranged from 0.058-0.065.

^1^Results are presented by Calus et al. [22].

**Table 3:** **Performance comparison of seven prediction methods in seven training scenarios for line B2**

	**Training data**						
**Model**	**B1**	**B2**	**W1**	**B1 + B2**	**B1 + W1**	**B2 + W1**	**B1 + B2 + W1**
GBLUP^1^	0.079	0.192	0.079	0.194	0.111	0.212	0.219
RRPCA^1^	0.091	0.286	0.070	0.304	0.118	0.296	0.316
MTGBLUP	0.080	0.223	−0.086	0.244	0.046	0.213	0.235
Poly	0.011	0.231	−0.083	0.231	−0.081	0.225	0.226
PolyPCA	0.002	0.230	−0.085	0.230	−0.083	0.224	0.224
RBF	0.063	0.232	0.083	0.236	0.068	0.233	0.237
RBFPCA	0.105	0.270	0.151	0.278	0.112	0.271	0.279

The predictive correlation is computed as the correlation coefficient of the predicted value and phenotype of line B2; GBLUP: genome-enabled best linear unbiased prediction; RRPCA: ridge regression principal component analysis; MTGBLUP: multi-trait GBLUP; Poly: polynomial kernel based linear models; RBF: radial basis function kernel based linear models; RR/Poly/RBF-PCA: the model with the features reduced by PCA.

Approximated SE across the genomic prediction models and training data sets ranged from 0.059-0.065.

^1^Results are presented by Calus et al. [22].

**Table 4:** **Performance comparison of seven prediction methods in seven training scenarios for line W1**

	**Training data**						
**Model**	**B1**	**B2**	**W1**	**B1 + B2**	**B1 + W1**	**B2 + W1**	**B1 + B2 + W1**
GBLUP^1^	−0.241	−0.115	0.547	−0.280	0.532	0.544	0.532
RRPCA^1^	−0.176	−0.177	0.551	−0.250	0.532	0.549	0.532
MTGBLUP	0.154	0.155	0.547	0.253	0.559	0.536	0.551
Poly	0.205	0.189	0.515	0.298	0.520	0.515	0.520
PolyPCA	0.207	0.190	0.515	0.299	0.521	0.515	0.521
RBF	−0.206	−0.089	0.530	−0.212	0.530	0.530	0.530
RBFPCA	−0.171	−0.149	0.540	−0.235	0.540	0.540	0.540

The predictive correlation is computed as the correlation coefficient of the predicted value and phenotype of line W1; GBLUP: genome-enabled best linear unbiased prediction; RRPCA: ridge regression principal component analysis; MTGBLUP: multi-trait GBLUP; Poly: polynomial kernel based linear models; RBF: radial basis function kernel based linear models; RR/Poly/RBF-PCA: the model with the features reduced by PCA.

Approximated SE across the genomic prediction models and training data sets ranged from 0.045-0.060.

^1^Results are presented by Calus et al. [22].

Table 2 shows the predictive correlations of line B1 across the training datasets. The impact of multi-line training for line B1 differed slightly between models. Results of the two models with the highest predictive correlations are discussed as examples. The GBLUP model achieved the highest predictive correlation when the model was trained exclusively on data from line B1. In other words, enlarging the training set by adding the training animals from any other line deteriorated the prediction performance. However, the second best model, namely RBF, which had a performance that was slightly inferior to that of the GBLUP model, benefited slightly from enhancing training with data from other lines.

Table 3 contains the predictive correlations for line B2. Compared to the scenario for which the training dataset only contained line B2, both linear models GBLUP and RRPCA had a ~0.03 higher predictive correlation with multi-line prediction. Predictive correlations for the non-linear models were, however, very similar to each other across the training datasets.

Interestingly, focussing on the results for line B1 with training on data from line B2 only, or vice versa, the predictive correlations of the linear and RBF models were clearly superior to those of the Poly models. This suggests that the genotypes of lines B1 and B2 shared some structural similarities that benefitted the predictions of the linear and RBF models. In these situations, the Poly models resulted in predictive correlations that were generally close to 0.

Table 4 shows the predictive correlations for the line W1 validation data. Predictive correlations were very similar across models and training datasets whenever line W1 was included in the training data. When line W1 was not included in the training data, the predictive correlations were always negative, except for MTGBLUP and the Poly models.

Overall, the benefit of multi-line training was limited, and only clearly observed in a few cases when the training data included a closely related line, e.g. lines B1 and B2. Therefore, enlarging the training set with unrelated or distantly related animals did not significantly improve predictive correlations.

### Bias of genomic prediction within and across lines

Bias of genomic predictions was assessed by evaluating coefficients of the regression of phenotypes on GEBV. Bias decreases as regression coefficients get closer to 1. For all three lines (See Additional file 1: Tables S1, S2 and S3), bias was more controlled for all models if the evaluated line was included in the training data, otherwise, large biases were observed, especially for the non-linear (Poly and RBF) models. These results indicate that GBLUP, RRPCA, MTGBLUP and RBFPCA gave reasonable results in terms of bias, as long as the evaluated line or a closely related line was included in the training dataset.

### Model comparison

Among the non-linear models, the Poly models generally performed worse than the RBF models, both in terms of predictive correlations (Tables 2, 3 and 4) and bias (See Additional file 1: Tables S1, S2 and S3), when the evaluated line was included in the training data. In addition, the predictions of the Poly models had close to 0 predictive correlations and very large biases when based on information from a closely related line (lines B1 and B2).

In the comparison between linear and non-linear models, it is important to note that the non-linear RBF models yielded predictive correlations that were comparable to those of the best linear models (either GBLUP or RRPCA) for lines B1 and W1 when the training data included all lines (Tables 2 and 4). For line B2, RBF performed better than the GBLUP model, while RRPCA had the highest predictive correlation in all scenarios (Table 3). For line B1, however, RRPCA had a lower predictive correlation than the RBF and GBLUP models (Table 2). For lines B1 and B2, the MTGBLUP model generally yielded predictive correlations that were similar to those of most of the other models (Tables 2 and 3). The same was observed for W1 when W1 was included in the training data (Table 4). However, when W1 was not included in the training data, MTGBLUP yielded positive predictive correlations but almost all other models yielded negative predictive correlations.

In summary, the results show that the performance of RBF models was fairly similar to that of the linear models, and that the Poly models generally performed worse. The MTGBLUP model in some situations could generate positive predictive correlations when the trait had a negative correlation between the evaluated line and the line(s) included in the training data.

### Complementarity analysis

**Figure 1:**
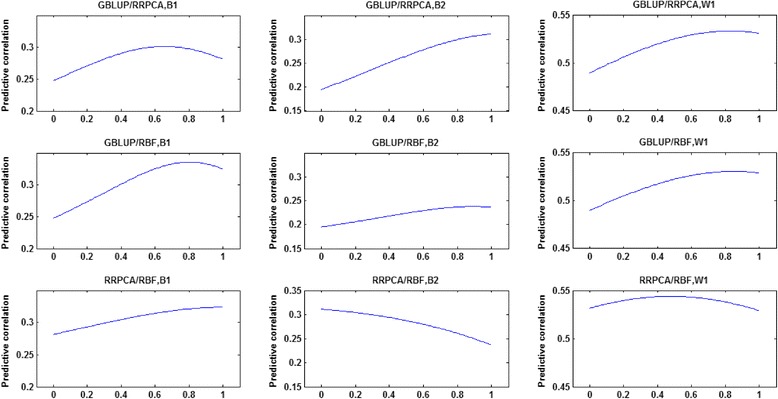
**Predictive correlations of weighted combinations of genomic predictions with the three models GBLUP, RRPCA, and RBF.** In each sub-figure i.e. “GBLUP/RRPCA, B1” means that the prediction is a combination of the predictions of models GBLUP and RRPCA and evaluated on line B1.

Because linear and non-linear models focus on different aspects of the genomic data, in this subsection, we analysed the complementarity between models. One way to measure the complementarity between two approaches is based on the correlation between their predictions. Correlations of genomic predictions were computed between models for the training dataset that included all three lines (Table 5). In general, predictions from the Poly models had the lowest correlations with those of other models, which is in line with the observation that, in most cases, the Poly models had the poorest performance in terms of predictive correlation. Ignoring the Poly models, the correlations between predictions from the different models were generally high (>0.9) for line W1. For lines B1 and B2, the predictions from the RBF models had correlations lower than 0.9 with those of GBLUP and RRPCA and higher than 0.9 with those of MTGBLUP. The prediction from the MTGBLUP model deviated substantially from those of GBLUP, with correlations of 0.91 to 0.98. The level of the correlations showed that combining predictions of different models could lead to more accurate predictions. The potential of such an approach was investigated by evaluating combined predictions of two models. A weighted combination of two predictions (*â*_1_, *â*_2_), can be easily obtained using the following equation:

$$ \widehat{a}=\beta {\widehat{a}}_1+\left(1-\beta \right){\widehat{a}}_2,0\le \beta \le 1, $$

where parameter *β* defines the weight given to the two approaches. When *β* is equal to 0 or 1, the combination is reduced to either of the two predictions. Figure 1 shows the predictive correlations of this combined prediction for the linear models GBLUP and RRPCA and the non-linear model RBF. In Figure 1, each row represents the results for one combination of models and each column represents the results for one of the lines. For line B1, combining predictions from a linear and a non-linear model improved the predictive correlation, especially for the combination of GBLUP and RBF. For line B2, there was little gain by combining models, which is probably due to the superior performance of the RRPCA model. For line W1, the combined prediction was in all cases slightly more accurate. Interestingly, across all situations, the benefit of combining predictions of two models was largest when the two models had a similar predictive correlation.

**Table 5:** **Correlation between genomic predictions obtained from the seven prediction methods**

**Line**	**Model**	**GBLUP**	**RRPCA**	**MTGBLUP**	**POLY**	**POLYPCA**	**RBF**
B1	RRPCA	0.877					
	MTGBLUP	0.913	0.900				
	POLY	0.677	0.751	0.796			
	POLYPCA	0.672	0.746	0.792	1.000		
	RBF	0.824	0.861	0.900	0.955	0.952	
	RBFPCA	0.781	0.870	0.861	0.901	0.897	0.957
B2	RRPCA	0.892					
	MTGBLUP	0.928	0.885				
	POLY	0.777	0.815	0.864			
	POLYPCA	0.774	0.812	0.863	1.000		
	RBF	0.867	0.883	0.928	0.968	0.966	
	RBFPCA	0.800	0.881	0.870	0.912	0.908	0.946
W1	RRPCA	0.964					
	MTGBLUP	0.975	0.965				
	POLY	0.921	0.871	0.905			
	POLYPCA	0.920	0.872	0.906	1.000		
	RBF	0.945	0.904	0.931	0.995	0.995	
	RBFPCA	0.905	0.903	0.894	0.963	0.963	0.969

Genomic predictions were obtained using all three lines in the training data.

### Computational complexity

For practical applications of genomic prediction in livestock, it is important that the predictions can be computed efficiently. Therefore, in this section, we analytically evaluate the computational complexity of linear and non-linear models. Revisiting both prediction models, they can be generalized by the following expression:

$$ {\mathbf{y}}^{*}={\mathbf{y}}^{\mathrm{t}}{\left(\mathbf{A}+\gamma \mathbf{I}\right)}^{-1}\mathbf{b}, $$

where **y** is the vector of training phenotypes. For the linear model, **A** = **XX**^*t*^ and **b** = **Xx**_*t*_ (referring to Equation (5)), while for the non-linear model **A** = **K** and **b** = **k** (referring to Equation (7)). The computation cost depends heavily on the inversion of matrix (**A** + *γ***I**)^− 1^, which is o(*n*^3^) [25]. Parameter *n* is equal to the dimension of matrix **A**. The computational complexity of the linear and non-linear models depends on the size of matrix **A**, which is *m* × *m* (i.e. ridge regression BLUP) or *n* × *n* (i.e. GBLUP) for the linear models and *n* × *n* for the non-linear models implemented in our study, which means that the complexities are either o(*m*^3^) or o(*n*^3^).

In genomic prediction, the number of genotypes (*m*) is typically much larger than the number of training animals (*n*). When ridge regression is used in the linear model (i.e. matrix **A** is of size *m* × *m*) and combined with the use of PCA (i.e. RRPCA in our case), the size of the matrix decreases to less than *n* × *n*, because the number of retained principal components will have a maximum value of *n*-1 [4]. Therefore, computational complexity of the non-linear models implemented in our study is comparable to that of the linear GBLUP model, as summarized in Table 6. Thus, the non-linear models are expected to be able to deal with datasets of similar size as the commonly used GBLUP model.

**Table 6:** **Computational complexity of the implemented linear and non-linear prediction models**

	**Linear models**		**Non-linear models**
	**Ridge regression**	**GBLUP**	
With PCA	o(*r* ^3^)	o(*n* ^3^)	o(*n* ^3^)
Without PCA	o(*m* ^3^)	o(*n* ^3^)	o(*n* ^3^)

For linear and non-linear models both implementations with and without PCA are considered. In the table, *r*, *m*, and *n* indicate the number of principal components (*r*), genotypes (*m*), and training animals (*n*). In the case of genomic prediction, generally *r* ≤ *n* ≪ *m*.

## Discussion

The objective of this study was to compare the accuracy of multi-line genomic prediction when using non-linear or linear models. In general, when the evaluated line was included in the training data, the non-linear RBF models yielded similar predictive correlations as the linear models. The non-linear models appeared to be slightly less sensitive to the structure of multi-line training datasets. For example, some of the linear models showed small decreases in predictive correlations for lines B1 and W1 when adding other lines [22], but this did not (or rarely) occur for the non-linear models. When only information from a closely related line was used for training, the linear models and the non-linear RBF models had similar performance, indicating that the strong assumptions of the linear models may at least partly hold for the closely related lines used in our study. Our expectation was that the non-linear models would be better able to use relevant information, without making strong assumptions as done in the linear models [21,39], but the results showed that, overall, the linear models and non-linear RBF models performed similarly.

The complementarity analysis is another aspect of our study. It has been shown that combining genomic predictions of different models, a procedure also known as “bagging” [40], may lead to more robust predictions with generally a higher accuracy [41] or at the very least result in similar accuracies as achieved with the underlying models [42]. In our study, except for line B2, for which RRPCA performed significantly better than any other model, both measures of complementarity indicated that combining linear and non-linear models has the potential to result in slightly more accurate predictions, which means that the linear models capture different features of the data than the non-linear models. The fact that non-linear models captured some predictive variation that was not explained by linear models may be partly due to the ability of non-linear models to capture non-additive effects. Since many non-additive effects are not passed onto the next generation, predictions from non-linear models may be less useful for achieving genetic gain than the linear models. Nevertheless, capturing non-additive effects does help to better predict the performance of an animal itself.

Another focus of this study was to investigate whether the potential benefit of multi-line genomic prediction depends on the genomic similarities of the lines considered. We showed that only some of the lines benefitted from multi-line training, which is consistent with previous studies e.g. [8,12]. The genotype data of the lines analysed in this work were apparently quite heterogeneous and thus, there was no consistent gain in predictive correlations from using multi-line training data. In some situations, there was a small benefit for lines B1 and B2 but not for W1. This was as expected based on results of the genotype-distance matrix reported by Calus et al. [22], that showed that animals from lines B1 and B2 were more closely related than animals from lines B1 or B2 with animals from line W1. Training data for which relationships with the predicted data are poor, are expected to have negligible contributions to the non-linear predictor. In contrast, the distance between two individuals from lines B1 and B2 was relatively small, indicating that the properties of the genotypes of these two lines were similar. These properties include allele frequencies and LD. Similarities between populations in both of these properties were shown to be closely related to genomic relationships between populations [43]. This might explain the improvement in predictive correlations for lines B1 and B2 in some scenarios when line B1 or B2 was added to the training data. Indeed, the estimated genetic correlations between the lines revealed that the trait investigated was highly correlated between lines B1 and B2. There was, however, no clear improvement in or even deterioration of predictive correlations for lines B1 and B2 when line W1 was included in training, or vice versa. However, across several linear models, positive predictive correlations of 0.10 to 0.14, although not significantly greater than 0, were consistently obtained for line B2 when only line W1 was used for training [22]. Moreover, genetic correlations were equal to −0.26 between lines B1 and W1 and −0.55 between lines B2 and W1, which suggests that information of line W1 was not useful for lines B1 and B2 and vice versa. In summary, a benefit from using multi-line training is especially expected when lines share several common properties, which can be characterized by genomic relationships between lines. Estimating the genetic correlation of the trait between lines may also be very informative. If the distance between the lines is very large and if the estimated correlation is close to 0 or even negative, the benefit of using multi-line genomic prediction is expected to be very limited.

Another interesting conclusion of the comparison between models for the three lines is that no single model was superior over all others for each scenario, which is similar to the results obtained when comparing different linear models [22]. The MTGBLUP model did not necessarily perform better than the other models for lines B1 and B2, but was able to yield substantial positive predictive correlations for line W1 when line B1, B2, or both were used for training. However, when line W1 was used to predict lines B1 and B2, MTGBLUP performed considerably worse than the other linear models. For predicting line B2, RRPCA performed much better than the other models. Interestingly, for line B2, the RBFPCA model was also more advantageous than the other regression models. For predicting line W1, all models performed quite similar whenever line W1 itself was included in the training data.

As an important criterion for model evaluation, the bias of the genomic predictions was evaluated (See Additional file 1: Tables S1, S2 and S3). First, when training and validation data were from the same line, the bias was limited for all models. The genotype distance between a brown hen and a white hen is relatively large such that the kernel value of those two genotypes by Equation (10) becomes small. Therefore, the variance of the GEBV becomes small and the bias accordingly can become very large. In other words, the non-linear models may yield realistic predictive correlations close to 0 combined with very large biases, while the strong assumptions of the linear model appear to control the bias, but at the same time may result in poor predictive correlations. These results highlight the importance of evaluating bias as well as accuracy if the predicted line or breed is not represented in the training data. Conversely, our results show that including the evaluated line in the training data is the best way to control the bias of the predictions, regardless of the model used.

By achieving a significant reduction in the dimension of genotypes, PCA is shown to benefit non-linear models, similar to what has been observed for the linear RRPCA model [22]. Concentrating on the non-linear kernel model that produced the highest predictive correlations, i.e. the RBF kernel, PCA had a minor impact on the predictive correlations, as shown by the correlation between the predictions from RBF and RBFPCA. This might be explained by the nature of the non-linear model: the prediction depends heavily on the distance relationships between training and testing animals, which are not altered by PCA. The Poly models also had very similar predictions whether PCA was performed or not. Regardless, the performance of Poly models was generally worse than that of other models, suggesting that they should not be considered for genomic prediction. Overall, our results with the non-linear RBF and linear RRPCA models suggest that dimensionality reduction of the genotype data might be helpful to decrease computational complexity while hardly affecting model accuracy.

## Conclusions

In this study, we investigated genomic prediction with multi-line data. Considering the possible complex heterogeneous data distributions of genotypes in such data, we used non-linear models by kernel linear regression, which rely on the similarity among animals but do not make assumptions on the linearity of genotypes, as the conventional linear models do. On this basis, it was anticipated that the non-linear models would capture different features of multi-line data than the linear models.

Our results indicate that the non-linear RBF models had very similar prediction performance as the generally used linear model GBLUP. Using one line to predict performance in another closely related line, yielded similar prediction accuracies with the RBF and the considered linear models, which suggests that the genotypes of closely related lines share some structural similarities. This was supported by the estimated genetic correlation of 0.63 between the trait in the two closely related lines. Using only data from a distantly related line for prediction with a linear model resulted sometimes in -small positive predictive correlations, in a few cases in considerable negative predictive correlations, and sometimes in predictions with very large bias. This suggests that genomic prediction using only information from a distantly related line or breed should be avoided. Furthermore, despite the similar predictive correlations, linear and non-linear models were shown to capture some complementary predictive information, since the combined prediction slightly improved the predictive correlations.

## Additional file

Additional file 1: Table S1. Coefficients of regression (RC) and their standard errors (SE) of observed phenotypes on predicted breeding values of seven different methods in seven training scenarios for line B1. Description: GBLUP: genome-enabled best linear unbiased prediction; RRPCA: ridge regression principal component analysis; MTGBLUP: multi-trait GBLUP; Poly: polynomial kernel based linear models; RBF: radial basis function kernel based linear models; RR/Poly/RBF-PCA: the model with the features reduced by PCA. **Table S2.** Coefficients of regression (RC) and their standard errors (SE) of observed phenotypes on predicted breeding values of seven different methods in seven training scenarios for line B2. Description: GBLUP: genome-enabled best linear unbiased Prediction; RRPCA: ridge regression principal component analysis; MTGBLUP: multi-trait GBLUP; Poly: pPolynomial kernel based linear models; RBF: radial basis function kernel based linear models; RR/Poly/RBF-PCA: the model with the features reduced by PCA. **Table S3.** Coefficients of regression (RC) and their standard errors (SE) of observed phenotypes on predicted breeding values of seven different methods in seven training scenarios for line W1. Description: GBLUP: genome-enabled best linear unbiased prediction; RRPCA: ridge regression principal component analysis; MTGBLUP: multi-trait GBLUP; Poly: polynomial kernel based linear models; RBF: radial basis function kernel based linear models; RR/Poly/RBF-PCA: the model with the features reduced by PCA.
