# Supplementary material for: Genomic prediction based on data from three layer lines using non-linear regression models
Source: Genet Sel Evol. 2014 Nov 6;46(1):75. doi: 10.1186/s12711-014-0075-3 (PMC4221696; doi:10.1186/s12711-014-0075-3)
Supplement: Additional file 1: Table S1. — Coefficients of regression (RC) and their standard errors (SE) of observed phenotypes on predicted breeding values of seven different methods in seven training scenarios for line B1. Description: GBLUP: genome-enabled best linear unbiased prediction; RRPCA: ridge regression principal component analysis; MTGBLUP: multi-trait GBLUP; Poly: polynomial kernel based linear models; RBF: radial basis function kernel based linear models; RR/Poly/RBF-PCA: the model with the features reduced by PCA. Table S2. Coefficients of regression (RC) and their standard errors (SE) of observed phenotypes on predicted breeding values of seven different methods in seven training scenarios for line B2. Description: GBLUP: genome-enabled best linear unbiased Prediction; RRPCA: ridge regression principal component analysis; MTGBLUP: multi-trait GBLUP; Poly: pPolynomial kernel based linear models; RBF: radial basis function kernel based linear models; RR/Poly/RBF-PCA: the model with the features reduced by PCA. Table S3. Coefficients of regression (RC) and their standard errors (SE) of observed phenotypes on predicted breeding values of seven different methods in seven training scenarios for line W1. Description: GBLUP: genome-enabled best linear unbiased prediction; RRPCA: ridge regression principal component analysis; MTGBLUP: multi-trait GBLUP; Poly: polynomial kernel based linear models; RBF: radial basis function kernel based linear models; RR/Poly/RBF-PCA: the model with the features reduced by PCA. [file 12711_2014_75_MOESM1_ESM.docx]

# Additional file 1

### Table S1 – Coefficients of regression (RC) and their standard errors (SE) of observed phenotypes on predicted breeding values of seven different methods in seven training scenarios for line B1.

|  | Training data | | | | | | | | | | | | | |
| --- | --- | --- | --- | --- | --- | --- | --- | --- | --- | --- | --- | --- | --- | --- |
|  | B1 | | B2 | | W1 | | B1+B2 | | B1+W1 | | B2+W1 | | B1+B2+W1 | |
| Model | RC | SE | RC | SE | RC | SE | RC | SE | RC | SE | RC | SE | RC | SE |
| GBLUP^1^ | 1.231 | 0.216 | 0.958 | 0.366 | -0.531 | 0.953 | 1.099 | 0.216 | 1.160 | 0.213 | 0.717 | 0.319 | 1.040 | 0.208 |
| RRPCA^1^ | 1.288 | 0.259 | 1.000 | 0.469 | 1.218 | 1.033 | 1.167 | 0.249 | 1.242 | 0.257 | 0.964 | 0.412 | 1.129 | 0.238 |
| MTGBLUP | 1.428 | 0.303 | 2.344 | 0.801 | -3.248 | 5.882 | 1.422 | 0.294 | 1.423 | 0.301 | 5.196 | 1.819 | 1.424 | 0.295 |
| Poly | 2.343 | 0.425 | -232.1 | 667.0 | 2.207 | 10.642 | 2.343 | 0.426 | 2.353 | 0.424 | 1.265 | 10.61 | 2.353 | 0.426 |
| PolyPCA | 2.372 | 0.436 | -631.0 | 995.1 | 2.001 | 9.593 | 2.372 | 0.437 | 2.383 | 0.433 | 0.988 | 9.584 | 2.383 | 0.437 |
| RBF | 1.761 | 0.298 | 6.458 | 2.085 | 5.189 | 55.947 | 1.752 | 0.293 | 1.760 | 0.300 | 6.375 | 2.085 | 1.750 | 0.294 |
| RBFPCA | 0.853 | 0.171 | 1.993 | 1.097 | 9.956 | 19.900 | 0.848 | 0.172 | 0.852 | 0.170 | 1.998 | 1.085 | 0.847 | 0.172 |

## ^1^ Results are presented by Calus et al. [[22](#_ENREF_22)].

GBLUP: Genome-enabled Best Linear Unbiased Prediction (GBLUP); RRPCA: Ridge Regression Principal Component Analysis; MTGBLUP: multi-trait GBLUP; Poly: Polynomial kernel based linear models; RBF: Radial Basis Function kernel based linear models; RR/Poly/RBF-PCA: the model with the features reduced by PCA.

### Table S2 – Coefficients of regression (RC) and their standard errors (SE) of observed phenotypes on predicted breeding values of seven different methods in seven training scenarios for line B2.

|  | Training data | | | | | | | | | | | | | |
| --- | --- | --- | --- | --- | --- | --- | --- | --- | --- | --- | --- | --- | --- | --- |
|  | B1 | | B2 | | W1 | | B1+B2 | | B1+W1 | | B2+W1 | | B1+B2+W1 | |
| Model | RC | SE | RC | SE | RC | SE | RC | SE | RC | SE | RC | SE | RC | SE |
| GBLUP^1^ | 0.383 | 0.306 | 0.696 | 0.217 | 0.893 | 0.689 | 0.623 | 0.199 | 0.501 | 0.285 | 0.752 | 0.207 | 0.701 | 0.194 |
| RRPCA^1^ | 0.594 | 0.396 | 1.153 | 0.234 | 0.966 | 0.838 | 1.188 | 0.232 | 0.675 | 0.340 | 1.149 | 0.230 | 1.195 | 0.226 |
| MTGBLUP | 0.876 | 0.651 | 1.008 | 0.268 | -2.411 | 1.802 | 1.082 | 0.275 | 0.646 | 0.816 | 0.982 | 0.279 | 1.060 | 0.276 |
| Poly | 75.5 | 487.4 | 1.669 | 0.419 | -8.831 | 6.663 | 1.668 | 0.419 | -8.730 | 6.646 | 1.650 | 0.419 | 1.651 | 0.421 |
| PolyPCA | 19.5 | 708.1 | 1.697 | 0.424 | -8.312 | 6.128 | 1.697 | 0.423 | -8.183 | 6.147 | 1.673 | 0.435 | 1.675 | 0.433 |
| RBF | 2.028 | 1.930 | 1.139 | 0.279 | 42.6 | 31.6 | 1.149 | 0.281 | 2.156 | 1.936 | 1.141 | 0.278 | 1.151 | 0.285 |
| RBFPCA | 1.597 | 0.961 | 0.724 | 0.158 | 35.7 | 14.3 | 0.749 | 0.157 | 1.683 | 0.961 | 0.726 | 0.157 | 0.751 | 0.157 |

## ^1^ Results are presented by Calus et al. [[22](#_ENREF_22)].

GBLUP: Genome-enabled Best Linear Unbiased Prediction (GBLUP); RRPCA: Ridge Regression Principal Component Analysis; MTGBLUP: multi-trait GBLUP; Poly: Polynomial kernel based linear models; RBF: Radial Basis Function kernel based linear models; RR/Poly/RBF-PCA: the model with the features reduced by PCA.

### Table S3 – Coefficients of regression (RC) and their standard errors (SE) of observed phenotypes on predicted breeding values of seven different methods in seven training scenarios for line W1.

|  | Training data | | | | | | | | | | | | | |
| --- | --- | --- | --- | --- | --- | --- | --- | --- | --- | --- | --- | --- | --- | --- |
|  | B1 | | B2 | | W1 | | B1+B2 | | B1+W1 | | B2+W1 | | B1+B2+W1 | |
| Model | RC | SE | RC | SE | RC | SE | RC | SE | RC | SE | RC | SE | RC | SE |
| GBLUP^1^ | -3.147 | 0.822 | -1.754 | 1.016 | 1.273 | 0.127 | -3.033 | 0.678 | 1.270 | 0.136 | 1.312 | 0.134 | 1.325 | 0.140 |
| RRPCA^1^ | -3.086 | 1.225 | -2.951 | 1.069 | 1.395 | 0.137 | -3.133 | 0.805 | 1.353 | 0.140 | 1.448 | 0.144 | 1.405 | 0.150 |
| MTGBLUP | 12.67 | 5.655 | 6.671 | 2.779 | 1.548 | 0.153 | 6.173 | 1.617 | 1.554 | 0.151 | 1.522 | 0.149 | 1.527 | 0.149 |
| Poly | 11.37 | 3.583 | 8.171 | 2.728 | 1.192 | 0.128 | 10.94 | 2.149 | 1.200 | 0.128 | 1.179 | 0.127 | 1.187 | 0.125 |
| PolyPCA | 10.40 | 3.221 | 7.411 | 2.435 | 1.198 | 0.128 | 9.986 | 1.945 | 1.206 | 0.128 | 1.183 | 0.128 | 1.192 | 0.126 |
| RBF | -185.5 | 57.2 | -59.7 | 46.0 | 1.387 | 0.144 | -119.1 | 36.0 | 1.386 | 0.143 | 1.388 | 0.146 | 1.388 | 0.145 |
| RBFPCA | -73.7 | 29.9 | -46.6 | 20.4 | 0.928 | 0.092 | -62.2 | 17.0 | 0.928 | 0.094 | 0.929 | 0.093 | 0.929 | 0.093 |

## ^1^ Results are presented by Calus et al. [[22](#_ENREF_22)].

GBLUP: Genome-enabled Best Linear Unbiased Prediction (GBLUP); RRPCA: Ridge Regression Principal Component Analysis; MTGBLUP: multi-trait GBLUP; Poly: Polynomial kernel based linear models; RBF: Radial Basis Function kernel based linear models; RR/Poly/RBF-PCA: the model with the features reduced by PCA.
